# Supplementary material for: Nutrient Uptake of Two Semidomesticated Jaltomata Schltdl. Species for Their Cultivation
Source: Plants (Basel). 2025 Apr 4;14(7):1124. doi: 10.3390/plants14071124 (PMC11991384; doi:10.3390/plants14071124)
Supplement: Supplementary file 1 [file plants-14-01124-s001.zip › plants-3492935-supplementary.pdf]

## Supplemental Material

**Table S1**. SPAD readings of *Jaltomata procumbens* and *Jaltomata tlaxcala*, cultivated at three electrical conductivity levels of nutritive solution. Colegio de Postgraduados, Campus Montecillo, Texcoco, State of Mexico. September 2020 – February 2021. Data taken at 17, 31, 45, and 57 dat, corresponding to vegetative, flowering, fructification, and harvesting stages, respectively.

| FV | SPAD readings | | | |
|---|---|---|---|---|
| EC (dS $m^{-1}$) | Vegetative | Flowering | Fructification | Harvesting |
| 1 | 57.18 a | 56.15 a | 58.15 b | 62.27 a |
| 2 | 57.12 a | 59.82 a | 61.57 a | 63.65 a |
| 3 | 57.22 a | 57.87 a | 61.08 ab | 62.62 a |

Abbreviation: EC, electrical conductivity. Different letters indicate statistical differences (Tukey, $p \leq 0.05$).

**Table S2**. Effect of the Sps x EC combination on the total dry matter of *Jaltomata procumbens* and *Jaltomata tlaxcala* in the vegetative stage. Colegio de Postgraduados, Campus Montecillo, Texcoco, State of Mexico. September 2020 – February 2021. Data taken between 17 and 129 dat.

| Vegetative stage | Sps x EC combination | | | | | |
|---|---|---|---|---|---|---|
| | Jp x 1 | Jp x 2 | Jp x 3 | Jt x 1 | Jt x 2 | Jt x 3 |
| TDM (g $plant^{-1}$) | 2.83 a | 3.17 a | 3.38 a | 1.94 a | 2.31 a | 2.67 a |

Abbreviations: Jp x 1, *J. procumbens* x 1 dS $m^{-1}$; Jp x 2, *J. procumbens* x 2 dS $m^{-1}$; Jp x 3, *J. procumbens* x 3 dS $m^{-1}$; Jt x 1, *J. tlaxcala* x 1 dS $m^{-1}$; Jt x 2, *J. tlaxcala* x 2 dS $m^{-1}$; Jt x 3, *J. tlaxcala* x 3 dS $m^{-1}$; EC, electrical conductivity; TDM, total dry matter. Different letters indicate statistical differences (Tukey, $p \leq 0.05$).

**Table S3**. Effect of the species on the plant structures of *Jaltomata procumbens* and *Jaltomata tlaxcala*. Colegio de Postgraduados, Campus Montecillo, Texcoco, State of Mexico. September 2020 – February 2021. Data taken between 17 and 129 dat.

| FV | Plant structures | | | |
|---|---|---|---|---|
| Species | SL | RL | SD | LA |
| | **Vegetative stage (17 dat)** | | | |
| *Jaltomata procumbens* | 24.91 a | 20.63 a | 5.88 a | 312.36 a |
| *Jaltomata tlaxcala* | 11.92 b | 18.40 a | 4.33 b | 253.95 a |
| | **Flowering stage (31 dat)** | | | |
| *Jaltomata procumbens* | 61.28 a | 30.57 a | 8.55 a | 1371.67 a |
| *Jaltomata tlaxcala* | 32.36 b | 28.06 a | 6.50 b | 881.22 b |
| | **Fructification stage (45 dat)** | | | |
| *Jaltomata procumbens* | 89.46 a | 40.94 a | 10.49 a | 2541.7 a |
| *Jaltomata tlaxcala* | 42.06 b | 35.73 a | 6.67 b | 1251.2 b |
| | **Harvesting stage (57 dat)** | | | |
| *Jaltomata procumbens* | 162.19 a | 53.73 a | 12.83 a | 5980.20 a |
| *Jaltomata tlaxcala* | 64.36 b | 47.49 a | 7.91 b | 2058.20 b |

Abbreviations: SL, stem length; RL, root length; SD, stem diameter; LA, leaf area; dat, days after transplant. Different letters indicate statistical differences (Tukey, $p \leq 0.05$).
